# Prospective associations of leucocyte subtypes and obesity with the risk of developing cutaneous malignant melanoma in the UK Biobank cohort

**DOI:** 10.1186/s12885-024-12344-0

**Published:** 2024-05-23

**Authors:** Sofia Christakoudi, Konstantinos K. Tsilidis, Elio Riboli

**Affiliations:** 1 Department of Epidemiology and Biostatistics, School of Public Health, Imperial College London, White City Campus, 90 Wood Lane, London, W12 0BZ UK; 2 Department of Inflammation Biology, School of Immunology and Microbial Sciences, King’s College London, London, UK; 3 Department of Hygiene and Epidemiology, University of Ioannina School of Medicine, Ioannina, Greece

**Keywords:** Neutrophils, Monocytes, Lymphocytes, Abdominal obesity, ABSI, Obesity, BMI, Cutaneous malignant melanoma

## Abstract

**Background:**

Obesity is associated with chronic low-grade inflammation, which is linked to cancer development. Abdominal obesity (a body mass index, ABSI), however, has unusually been associated inversely with cutaneous malignant melanoma (CMM), while general obesity (body mass index, BMI) is associated positively. Leucocytes participate in inflammation and are higher in obesity, but prospective associations of leucocytes with cutaneous malignant melanoma are unclear.

**Methods:**

We examined the prospective associations of neutrophil, lymphocyte, and monocyte counts (each individually), as well as the prospective associations of ABSI and BMI, with cutaneous malignant melanoma in UK Biobank. We used multivariable Cox proportional hazards models and explored heterogeneity according to sex, menopausal status, age (≥ 50 years at recruitment), smoking status, ABSI (dichotomised at the median: ≥73.5 women; ≥79.8 men), BMI (normal weight, overweight, obese), and time to diagnosis.

**Results:**

During a mean follow-up of 10.2 years, 2174 CMM cases were ascertained in 398,450 participants. There was little evidence for associations with neutrophil or lymphocyte counts. Monocyte count, however, was associated inversely in participants overall (HR = 0.928; 95%CI: 0.888–0.971; per one standard deviation increase; SD = 0.144*10^9^/L women; SD = 0.169*10^9^/L men), specifically in older participants (HR = 0.906; 95%CI: 0.862–0.951), and more clearly in participants with low ABSI (HR = 0.880; 95%CI: 0.824–0.939), or with BMI ≥ 25 kg/m^2^ (HR = 0.895; 95%CI: 0.837–0.958 for overweight; HR = 0.923; 95%CI: 0.848–1.005 for obese). ABSI was associated inversely in pre-menopausal women (HR = 0.810; 95%CI: 0.702–0.935; SD = 4.95) and men (HR = 0.925; 95%CI: 0.867–0.986; SD = 4.11). BMI was associated positively in men (HR = 1.148; 95%CI: 1.078–1.222; SD = 4.04 kg/m^2^). There was little evidence for heterogeneity according to smoking status. The associations with monocyte count and BMI were retained to at least 8 years prior to diagnosis, but the association with ABSI was observed up to 4 years prior to diagnosis and not for longer follow-up time.

**Conclusions:**

Monocyte count is associated prospectively inversely with the risk of developing CMM in older individuals, while BMI is associated positively in men, suggesting a mechanistic involvement of factors related to monocytes and subcutaneous adipose tissue in melanoma development. An inverse association with ABSI closer to diagnosis may reflect reverse causality or glucocorticoid resistance.

**Supplementary Information:**

The online version contains supplementary material available at 10.1186/s12885-024-12344-0.

## Background

Cutaneous malignant melanoma (CMM) is the most serious and lethal skin cancer, which affects predominantly populations of European ancestry, and its incidence and mortality are projected to increase with more than 50% by 2040 [1]. We have previously shown that CMM has an unusual association pattern with general and abdominal obesity, showing a positive association with general obesity evaluated with body mass index (BMI), but an inverse association with abdominal obesity evaluated with the allometric body shape index (ABSI) [2], which is uncorrelated with BMI and defines abdominal obesity independently of general obesity [3]. An inverse association with ABSI is mechanistically plausible because activation of melanocortin receptors has been associated with CMM development [4], while their inactivation has been associated with development of obesity and metabolic syndrome [5]. A hallmark of obesity, and more specifically of abdominal obesity, is chronic low-grade inflammation [6], which is associated with cancer development [7]. Given the discordant associations of BMI and ABSI with CMM and the fact that white blood cell (leucocyte) counts were associated positively with both BMI and ABSI [8], a question emerges whether obesity-related chronic inflammation, as reflected in leucocyte subtype counts, is associated with the risk of developing CMM.

In this study, we have used data from the UK Biobank cohort to investigate the prospective associations of neutrophil, lymphocyte, and monocyte counts with the risk of developing CMM and have explored heterogeneity according to sex, menopausal status, age, smoking, abdominal obesity, and general obesity. In addition, we have extended the investigations of the associations of ABSI and BMI with CMM, with additional follow-up time and almost twice as many cases compared to our previous report [2], and have explored heterogeneity according to time to diagnosis.

## Methods

### Study population

UK Biobank is a prospective cohort, including half a million participants aged 40 to 70 years at recruitment (between 2006 and 2010), which were living within 40 km of an assessment centre in England, Scotland, and Wales, and were registered with the National Health Service [9]. In this study, we included participants with self-reported white ancestry (due to limited numbers from other ethnic groups) and excluded participants with missing or extreme anthropometric measurements, mismatch between the genetic and self-reported sex, pregnant women, and participants with prevalent cancer at recruitment, similarly to our previous report [2], but additionally excluding all prevalent skin cancers (CMM and non-melanoma skin cancers − basocellular and squamous cell) because we were interested in the prospective associations with CMM, i.e. incident CMM in individuals with no prior personal history of cancer for which the assessment of the exposure precedes the outcome. We further excluded participants with missing or extreme neutrophil, lymphocyte, or monocyte counts (Table 1). In total, we excluded 103,919 participants, 20.7% from the total available dataset, from which participants withdrawing consent by the time of analysis had already been excluded.

**Table 1:** Exclusion criteria

		Total	Women	Men
	Total available ^**a**^	502,369	273,301	229,068
1.	Ethnic background (restricted to self-reported white) ^**b**^	29,797	15,935	13,862
2.	Anthropometric measurements missing or extreme ^**c**^	7131	4758	2373
3.	Genetic & self-reported sex mismatch, or sex chromosome aneuploidy, or age < 40 or > 70 years, or pregnant at recruitment ^**b**^	898	493	405
4.	Prevalent cancer at recruitment ^**b**^	43,963	27,099	16,864
5.	Missing neutrophil, lymphocyte, or monocyte counts	19,235	11,161	8074
6.	Extreme leucocyte counts ^**d**^	2895	1560	1335
	Total excluded:	103,919	61,006	42,913
	(% from the available dataset)	(20.7)	(22.3)	(18.7)
	Total included:	398,450	212,295	186,155

The exclusion criteria were applied sequentially in the displayed order, counting each excluded individual only once

^**a**^ – participants withdrawing consent by the time of analysis were excluded from the total available

^**b**^ – for UK Biobank field names, definition of variables, and definition of prevalent cancer cases see Supplementary Methods in [2] and note that, for this study, we excluded all prevalent non-melanoma skin cancers (code 173 in the 9th version of the International Statistical Classification of Diseases (ICD9), or code C44 in ICD10, or self-reported), in addition to all prevalent cutaneous malignant melanomas (code 172 in ICD9, or code C43 in ICD10, or self-reported) and all prevalent cancers in other locations

^**c**^ – missing anthropometric measurements; height < 130 cm; waist circumference < 50 or > 160 cm; body mass index (BMI) < 18.5 or ≥ 45 kg/m^2^. Field names for waist and hip circumferences, weight, and height are listed in Supplementary Methods of [2]

^**d**^ – defined as values above the top 0.25^th^ sex-specific centile (neutrophils > 10.19 for women > 10.50 for men; lymphocytes > 4.48 for women > 4.39 for men; monocytes > 1.16 for women > 1.33 for men; unit *10^9^/L)

### Ascertainment of cutaneous malignant melanoma

UK Biobank is linked to the national cancer registry of the United Kingdom. The outcome of interest in this study was first primary CMM diagnosed after recruitment, with code C43 from the 10th version of the International Statistical Classification of Diseases (ICD10), histological codes within the range 8720 to 8790, and behavioural codes 3 (malignant, primary site) or 5 (malignant, microinvasive), as defined in [2]. Follow-up was censored at the date of diagnosis for participants with first incident CMM marked only with behavioural code 6 (malignant, metastatic site) or 9 (malignant, uncertain whether primary or metastatic site) or with missing behavioural code, or with non-melanoma skin cancer (code C44), or with cancer in location other than the skin, as for them CMM would not be the first primary cancer, they would have shown higher susceptibility to cancer development than the rest of the population, and would have undergone cancer treatment which is likely to affect the risk of subsequent cancer development. Follow-up was censored at the earlier of the date of death or the date of the last complete cancer registry (31st March 2020 for England and Scotland, 31st December 2016 for Wales) for all participants remaining free of cancer.

### Haematological and anthropometric measurements

Blood samples were collected irrespective of fasting status throughout the day in ethylenediaminetetraacetic acid (EDTA) vacutainers and were analysed within 24 h of blood draw on Beckman Coulter LH750 automated analysers [10]. Anthropometric measurements were obtained by trained technicians according to established protocols [11]. Waist circumference (WC) was measured at the natural indent or the umbilicus. We calculated ABSI according to [3] as WC(m)∗Weight(kg)^−2/3^∗Height(m)^5/6^, additionally multiplied by 1000, and calculated BMI as Weight(kg)∗Height(m)^−2^.

### Definition and selection of covariates

We considered several potential confounders selected *a priori* based on literature reports of their associations with the exposures and the outcome (Supplementary Table S1). The following covariates were defined as previously described in [2]: region of the assessment centre (London, North-West, North-East, Yorkshire and Humber, West Midlands, East Midlands, South-East, South-West, Wales, Scotland), weight change within the year preceding recruitment (weight loss, stable weight, weight gain), alcohol consumption (≤ 3 times/month, ≤ 4 times/week, daily), physical activity (less active, moderately active, very active), family history of cancer (lung/bowel/prostate/breast cancer in parents or siblings, no/yes), and sun-exposure-related factors, including skin colour (very fair; fair; dark), ease of skin tanning (very tanned; moderately tanned; mild/occasionally tanned; never tan/only burn), hair colour (blond/red; light brown; dark), childhood sunburns (never; ever; missing), solarium use (never/ever), sun/UV protection (never/rarely; sometimes; most of the time; always/do not go out in sunshine), and time spent outdoors in summer (≤ 3 h/day; >3 h/day; missing). Menopausal status (pre-menopausal, post-menopausal, unknown) and hormone replacement therapy (HRT) use (never, past, current) were defined similarly to [12]. Detailed categories for smoking status and intensity were defined according to [13]: never smoked; just tried; former occasional smoker; former regular quit ≥ 20 years; former regular quit ≥ 10 years; former regular quit < 10 years; current occasional smoker; current regular ≤ 10 cigarettes/day; current regular > 10 cigarettes/day. The following covariates were defined as previously described in [8]: time of blood collection (< 12:00; 12:00 to < 16:00; ≥16:00), fasting time (0–2 h; 3–4 h; ≥5 h), self-reported diabetes (no/yes, including all participants reporting use of anti-diabetic drugs and assuming that all participants with self-reported diabetes were treated), use of lipid lowering drugs (additionally including cholestyramine products), and antihypertensive drugs. Use of non-steroidal anti-inflammatory drugs (NSAID) and antiaggregant/anticoagulants was defined according to [13]. Use of any medication within the corresponding category was coded as yes, such that all variables of drug use were binary (no/yes). As a proxy for socioeconomic status, we used Townsend deprivation index (sex-specific quintiles).

We examined the associations of all candidate covariates, each individually, with BMI, ABSI, and leucocyte counts as outcomes (sex-specific z-scores, value minus mean, divided by standard deviation, SD) in linear regression models (adjusted for age and region of the assessment centre) and with CMM in Cox proportional hazards models (stratified by age, region, and sex) (Supplementary Table S2) and derived the final set of confounders (Supplementary Figure S1). We omitted use of NSAIDs, antiaggregant/anticoagulants, and lipid-lowering drugs, diabetes, weight change, and physical activity because there was little evidence for their association with CMM (Supplementary Table S2) and because diabetes and use of lipid-lowering drugs, as metabolic consequences of abdominal obesity, could act as mediators. Time of blood collection and fasting time offered a way of accounting for the collection of blood samples throughout the day and irrespective of fasting time but did not improve materially the precision of the estimates, so we omitted them from the final models. Sun-exposure-related factors were included in the final models because they showed associations with at least one of the exposures, in addition to CMM (Supplementary Table S2). To account for sex and female-specific characteristic in the final models, we used a combined variable reflecting sex, menopausal status, and HRT use, defined similarly to [12], with five categories: men; pre-menopausal women; post/unknown menopause never HRT; post/unknown menopause past HRT; post/unknown menopause current HRT.

### Statistical analysis

We used STATA-13 for the statistical analyses and R version 4.1.3 [14] for data management. Tests of statistical significance were two-sided.

We considered as main exposures neutrophil, lymphocyte, and monocyte counts, each examined individually on a standardised continuous scale (sex specific z-scores). We additionally considered as exposures ABSI and BMI (sex-specific z-scores). We interpreted hazard ratios (HR) per one SD increase in the exposure. We obtained HRs and 95% confidence intervals (95%CI) from delayed-entry multivariable Cox proportional hazards models, which account for left-truncation and are conditional on surviving free of cancer to cohort recruitment. We used age as the underlying time scale, with origin at the date of birth, entry time at the date at recruitment, and exit time at the earliest of the date of diagnosis of the first primary incident cancer, or death, or end of cancer follow-up.

For each individual exposure, we first examined four models with different adjustments: Model 1 – unadjusted and unstratified; Model 2 – stratified by age at recruitment (five-year categories), region of the assessment centre, and the combined variable reflecting sex, menopausal status, and HRT use, and adjusted for BMI (when considering ABSI as exposure) or ABSI (when considering BMI as exposure) and height (sex-specific z-scores), smoking status and intensity, alcohol consumption, Townsend deprivation index, family history of cancer, and use of antihypertensive drugs; Model 3 – like Model 2, with additional adjustment for sun-exposure-related factors (skin colour, ease of skin tanning, hair colour, childhood sunburns, solarium use, sun/UV protection, and time spent outdoors in summer); Model 4 (fully-adjusted) – like Model 3, with additional adjustment for BMI and ABSI (when examining leucocyte counts as exposures) or with additional adjustment for monocyte count (when examining ABSI and BMI as exposures), as this was the only leucocyte subtype showing clear associations with CMM. We replaced missing values for covariates with missingness < 5% with the median category for each sex and retained separate missing categories for childhood sunburns (24.7% missing) and time spent outdoors (5.4% missing), as in [2]. The proportion of missing values for each covariate are shown in Supplementary Table S3. In a sensitivity analysis, we repeated the fully adjusted model (Model 4) using multiple imputations for covariates with missingness > 1% (skin colour, ease of skin tanning, sunburns in childhood, and time spent outdoors in summer) [15].

For all exposures, we used the fully adjusted model (Model 4) to explore heterogeneity according to sex, menopausal status in women, and age in men (dichotomised at ≥ 50 years at recruitment). For monocyte count, we additionally examined heterogeneity according to age in participants overall, smoking status (never, former, current smokers), ABSI (dichotomised at the median, ≥ 73.531 for women, ≥ 79.763 for men), and BMI (normal weight: BMI = 18.5 to < 25; overweight: BMI = 25 to < 30, obese: BMI = 30 to < 45, unit kg/m^2^). For ABSI and BMI, we additionally examined heterogeneity according to smoking status. We compared HR estimates between groups with the data augmentation method of Lunn and McNeil [16]. We could not use multiple imputations for group comparisons, because these require a likelihood ratio test, which is not defined in the context of multiple imputations [15].

To examine heterogeneity according to time to diagnosis, we similarly used the data augmentation method of Lunn and McNeil [16] and compared HR estimates for three follow-up periods: <4 years; 4 to < 8 years; and ≥ 8 years post recruitment. Entry time was the date at the start of the corresponding follow-up period and exit time was the earliest of the date at diagnosis of the first primary incident cancer, or death, or the end of the corresponding follow-up period, or the end of cancer follow-up. Participants with first primary incident cancer or death prior to entry time for the corresponding follow-up period were excluded from the corresponding analysis.

## Results

### Cohort characteristics

During a mean follow-up of 10.2 years (SD = 2.40), 2174 CMM cases were ascertained in 398,450 participants. Neutrophil and monocyte counts were lower in women compared to men and in younger compared to older men, but neutrophil count was higher in pre-menopausal compared to post-menopausal women (Table 2). Lymphocyte count, on the contrary, was higher in women compared to men, but lower in pre-menopausal compared to post-menopausal women, with no material difference according to age in men. ABSI and BMI were higher in men compared to women, and in post-menopausal women and older men compared to pre-menopausal women and younger men, correspondingly. Participant characteristics with respect to covariates were consistent with our previous reports [2, 8, 12] (Supplementary Table S3).

**Table 2:** Leucocyte counts and obesity indices of study participants

Group	Cohort ^a^	Neutrophil count ^b^	Lymphocyte count ^b^	Monocyte count ^b^	ABSI ^b^	BMI ^b^
**Overall**	398,450	4.22 (1.34)	1.93 (0.58)	0.472 (0.161)	76.6 (5.5)	27.3 (4.5)
**Sex**						
Women	212,295 (53.3)	4.19 (1.32)	1.99 (0.59)	0.436 (0.144)	73.8 (5.0)	26.9 (4.8)
Men	186,155 (46.7)	4.26 (1.35)	1.87 (0.57)	0.513 (0.169)	79.8 (4.1)	27.8 (4.0)
p _sex_		< 0.0001	< 0.0001	< 0.0001	< 0.0001	< 0.0001
**Menopause**						
Pre-MP	51,520 (24.3)	4.44 (1.39)	1.91 (0.55)	0.437 (0.147)	72.5 (4.7)	26.2 (4.8)
Post-MP	137,046 (64.6)	4.08 (1.27)	2.01 (0.59)	0.436 (0.143)	74.2 (5.0)	27.0 (4.7)
p _menopause_		< 0.0001	< 0.0001	0.190	< 0.0001	< 0.0001
**Age (Men)**						
<50 years	43,408 (23.3)	4.08 (1.36)	1.88 (0.55)	0.480 (0.156)	78.2 (3.9)	27.5 (4.1)
≥50 years	142,747 (76.7)	4.31 (1.35)	1.86 (0.57)	0.523 (0.171)	80.2 (4.0)	27.9 (4.0)
p _age (men)_		< 0.0001	< 0.0001	< 0.0001	< 0.0001	< 0.0001

**ABSI** – a body shape index; **BMI** – body mass index; **MP** – menopause

^**a**^ number (percent from the total cohort (for Sex), or from total women (for Menopause), or from total men (for Age)); ^**b**^ mean (standard deviation)

Comparisons between sexes, menopausal groups (women), and age groups (men) were performed with unpaired-samples t-test

### Associations of leucocyte counts with cutaneous malignant melanoma

**Fig. 1:**
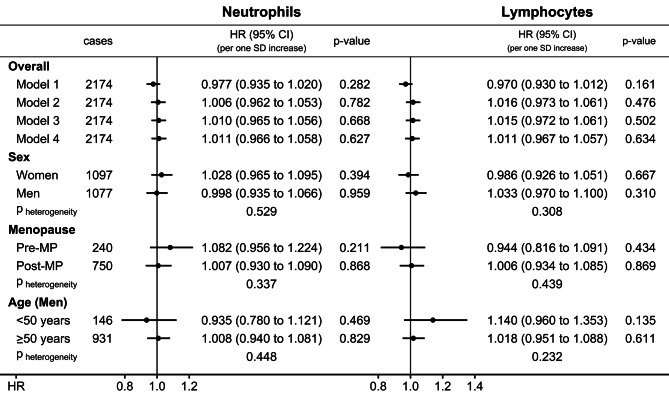
Prospective associations of neutrophil and lymphocyte counts with cutaneous malignant melanoma. **CI** – confidence interval; **HR** – hazard ratio; **MP** – menopause; **p-value** – Wald test for the individual term; **SD** – standard deviation. **Model 1** – unstratified and unadjusted Cox proportional hazards model for participants overall, with exposure either neutrophil or lymphocyte count (sex-specific z-scores, value minus mean divided by SD), with mean (SD) for neutrophils: 4.186 (1.317) for women, 4.258 (1.355) for men; and mean (SD) for lymphocytes: 1.990 (0.587) for women, 1.865 (0.565) for men. **Model 2** – like Model 1, stratified by age at recruitment, region, sex, and for analyses including women, menopausal status and hormone replacement therapy use, and adjusted for height (sex-specific z-scores), smoking status and intensity, alcohol consumption, Townsend deprivation index, family history of cancer, and use of antihypertensive drugs. **Model 3** – like Model 2, additionally adjusted for sun-exposure-related factors (skin colour, ease of skin tanning, hair colour, childhood sunburns, solarium use, sun/UV protection, and time spent outdoors in summer). **Model 4** – like Model 3, additionally adjusted for body mass index (BMI) and the allometric body shape index (ABSI) (sex-specific z-scores), used for all subgroup analyses. **p**_**heterogeneity**_ – p-value obtained with the data augmentation method of Lunn and McNeil [16] for the comparison of HR estimates between the specified groups according to sex, menopausal status (women), and age (men)

Neither neutrophil nor lymphocyte counts were associated materially with CMM, irrespective of stratification and adjustment, except for a possible positive association with lymphocyte count in younger men (HR = 1.140; 95%CI = 0.960–1.353) (Fig. 1).

**Fig. 2:**
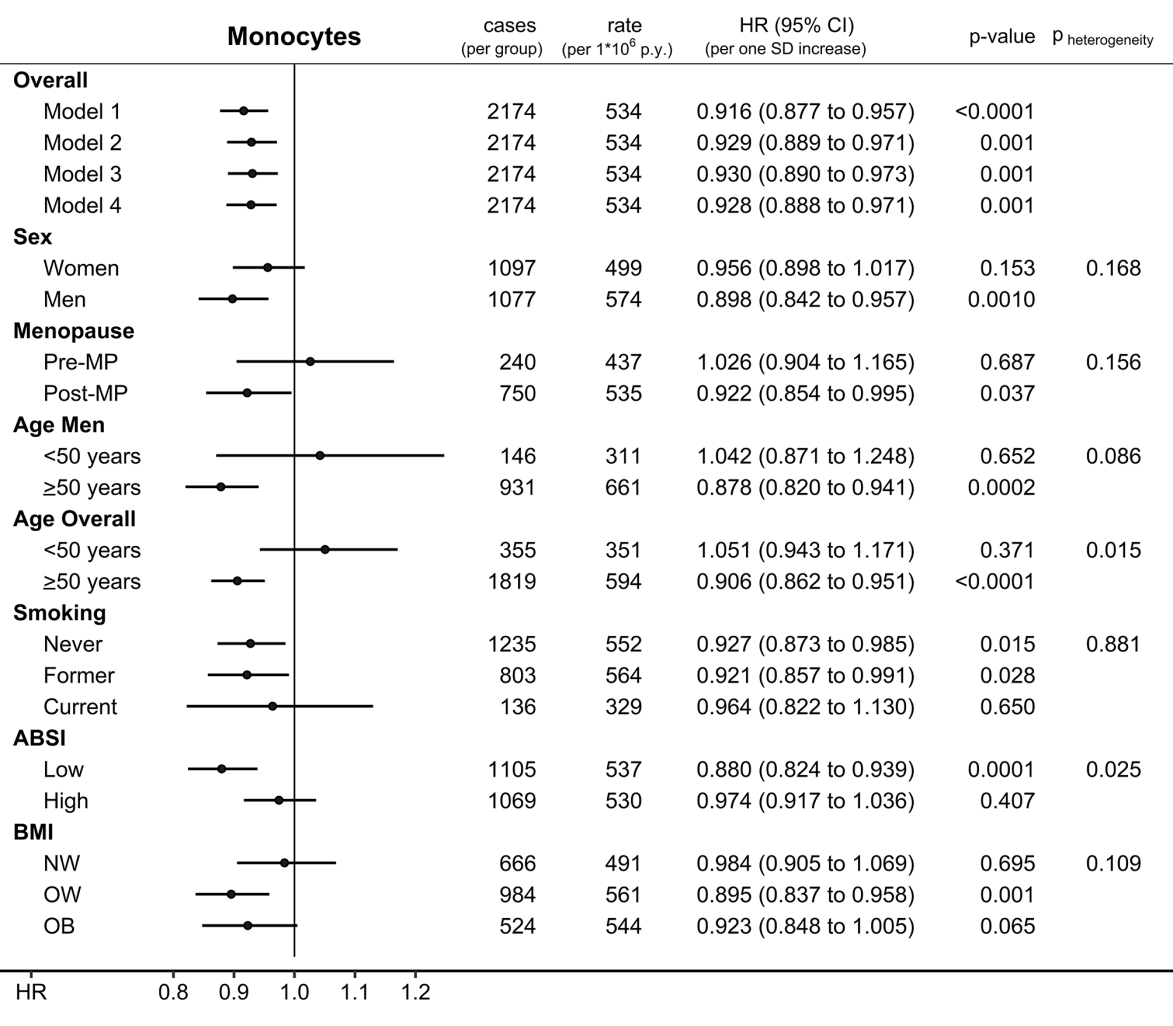
Prospective associations of monocyte count with cutaneous malignant melanoma. **ABSI** – a body shape index; **BMI** – body mass index; **cases** – number of cutaneous malignant melanoma cases per group; **CI** – confidence interval; **HR** – hazard ratio; **MP** – menopause; **NW** – normal weight (BMI = 18.5 to < 25 kg/m^2^); **OW** – overweight (BMI = 25 to < 30 kg/m^2^); **OB** – obese (BMI = 30 to < 45 kg/m^2^); **p-value** – Wald test for the individual term; **rate** – number of cutaneous malignant melanoma cases per 1,000,000 person years of follow-up; **SD** – standard deviation. **Model 1** – unstratified and unadjusted Cox proportional hazards model for participants overall with exposure monocyte count (sex-specific z-scores, value minus mean (0.436 for women; 0.513 for men) divided by SD (0.144 for women; 0.169 for men), unit *10^9^/L). **Model 2** – like Model 1, stratified by age at recruitment, region, sex, and for analyses including women, menopausal status and hormone replacement therapy use, and adjusted for height (sex-specific z-scores), smoking status and intensity, alcohol consumption, Townsend deprivation index, family history of cancer, and use of antihypertensive drugs. **Model 3** – like Model 2, additionally adjusted for sun-exposure-related factors (skin colour, ease of skin tanning, hair colour, childhood sunburns, solarium use, sun/UV protection, and time spent outdoors in summer). **Model 4** – like Model 3, additionally adjusted for BMI and ABSI (sex-specific z-scores), used for all subgroup analyses. **p**_**heterogeneity**_ – p-value obtained with the data augmentation method of Lunn and McNeil [16] for the comparison of HR estimates between the specified groups according to sex, menopausal status (women), age (men and overall), smoking status, BMI (NW vs. OW/OB), and ABSI (dichotomised at the sex-specific median: ≥73.531 for women; ≥79.763 for men)

Monocyte count was associated inversely with CMM, with little difference between HR estimates obtained without adjustment (HR = 0.916; 95%CI: 0.877–0.957 from Model 1) and with full adjustment, hence independent of ABSI, BMI, smoking, and other covariates (HR = 0.928; 95%CI: 0.888–0.971 from Model 4 per one SD increase: SD = 0.144*10^9^/L women; SD = 0.169*10^9^/L men) (Fig. 2). The estimates of the final model were similar when multiple imputations were used for missing values (Supplementary Table S4). The inverse association with monocyte count was directionally consistent in women and men but was noted more specifically in post-menopausal women and older men and not in younger participants (HR = 0.906; 95%CI = 0.862–0.951 for all participants aged ≥ 50 years at recruitment; p_heterogeneity_=0.015 for age in participants overall). There was no evidence for heterogeneity according to smoking status, although there were only 136 CMM cases in current smokers. The inverse association with monocyte count, however, was more prominent for ABSI below the sex-specific median (HR = 0.880; 95%CI = 0.824–0.939, p_heterogeneity_=0.025) and for overweight (HR = 0.895; 95%CI = 0.837–0.958) and obese participants (HR = 0.923; 95%CI = 0.848–1.005; p_heterogeneity_=0.109 comparing normal weight and overweight/obese).

### Associations of obesity indices with cutaneous malignant melanoma

**Fig. 3:**
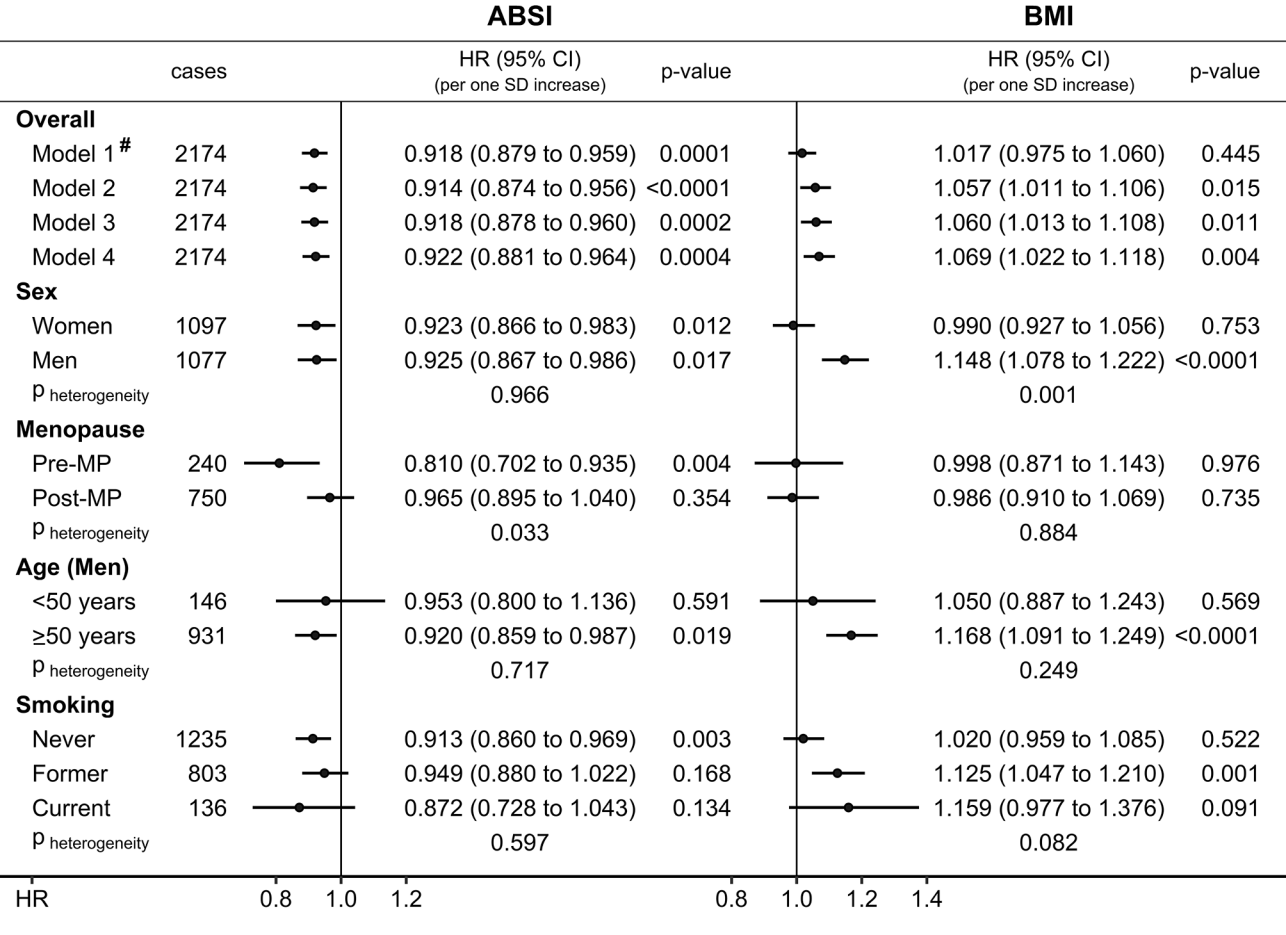
Prospective associations of obesity indices with cutaneous malignant melanoma. **ABSI** – a body shape index; **BMI** – body mass index; **cases** – number of cutaneous malignant melanoma cases per group; **CI** – confidence interval; **HR** – hazard ratio; **MP** – menopause; **p-value** – Wald test for the individual term; **SD** – standard deviation. **Model 1** – unstratified and unadjusted Cox proportional hazards model for participants overall, including either ABSI or BMI as exposure (sex-specific z-scores, value minus mean divided by SD), with mean (SD) for ABSI: 73.779 (4.953) for women, 79.756 (4.107) for men; and mean (SD) for BMI: 26.917 (4.779) for women, 27.804 (4.037) for men (unit kg/m^2^). **Model 2** – like Model 1, including jointly ABSI and BMI, stratified by age at recruitment, region, sex, and for analyses including women, menopausal status and hormone replacement therapy use, and adjusted for height (sex-specific z-scores), smoking status and intensity, alcohol consumption, Townsend deprivation index, family history of cancer, and use of antihypertensive drugs. **Model 3** – like Model 2, additionally adjusted for sun-exposure-related factors (skin colour, ease of skin tanning, hair colour, childhood sunburns, solarium use, sun/UV protection, and time spent outdoors in summer). **Model 4** – like Model 3, additionally adjusted for monocyte count (sex-specific z-scores), used for all subgroup analyses. **p**_**heterogeneity**_ – p-value obtained with the data augmentation method of Lunn and McNeil [16] for the comparison of HR estimates between the specified groups according to sex, menopausal status (women), age (men), and smoking status. ^**#**^ – adjustment of BMI for smoking status and intensity alone made no material difference to association estimates (HR = 1.021; 95%CI = 0.978–1.065; *p* = 0.343)

Like monocyte count, ABSI was clearly associated inversely with CMM, with little difference between HR estimates obtained without adjustment (HR = 0.918; 95%CI: 0.879–0.959 from Model 1) and with full adjustment, hence independent of monocyte count, smoking, and other covariates (HR = 0.922; 95%CI: 0.881–0.964 from Model 4 per one SD increase: SD = 4.95 women; SD = 4.11 men) (Fig. 3). Although there was no material difference between women and men overall, the inverse association with ABSI was noted only in pre-menopausal women (HR = 0.810; 95%CI: 0.702–0.935; p_heterogeneity_=0.033 for menopausal status), with no evidence for heterogeneity according to age in men.

BMI was not associated with CMM in the unadjusted model (HR = 1.017; 95%CI = 0.975–1.060 for Model 1) (Fig. 3). Adjustment only for smoking status and intensity made no material difference to association estimates (HR = 1.021; 95%CI = 0.978–1.065), but a positive association was noted after adjustment for all lifestyle factors (HR = 1.057; 95%CI: 1.011–0.106 from Model 2), with no material influence of further adjustment for sun-exposure-related factors, and little influence of additional adjustment for monocyte count (HR = 1.069; 95%CI: 1.022–1.118 from Model 4 per one SD increase: SD = 4.78 kg/m^2^ women; SD = 4.04 kg/m^2^ men). There was a clear sex difference, with a positive association with BMI only in men (HR = 1.148; 95%CI: 1.078–1.222) and not in women (p_heterogeneity_=0.001 for sex). Similar estimates were obtained from the final models when using multiple imputations for missing values (Supplementary Table S4).

There was no evidence for heterogeneity of the associations with ABSI according to smoking status, but the positive association with BMI was not observed in never smokers (p_heterogeneity_=0.082 for smoking status) (Fig. 3).

### Heterogeneity of the associations with cutaneous malignant melanoma according to time to diagnosis

**Fig. 4:**
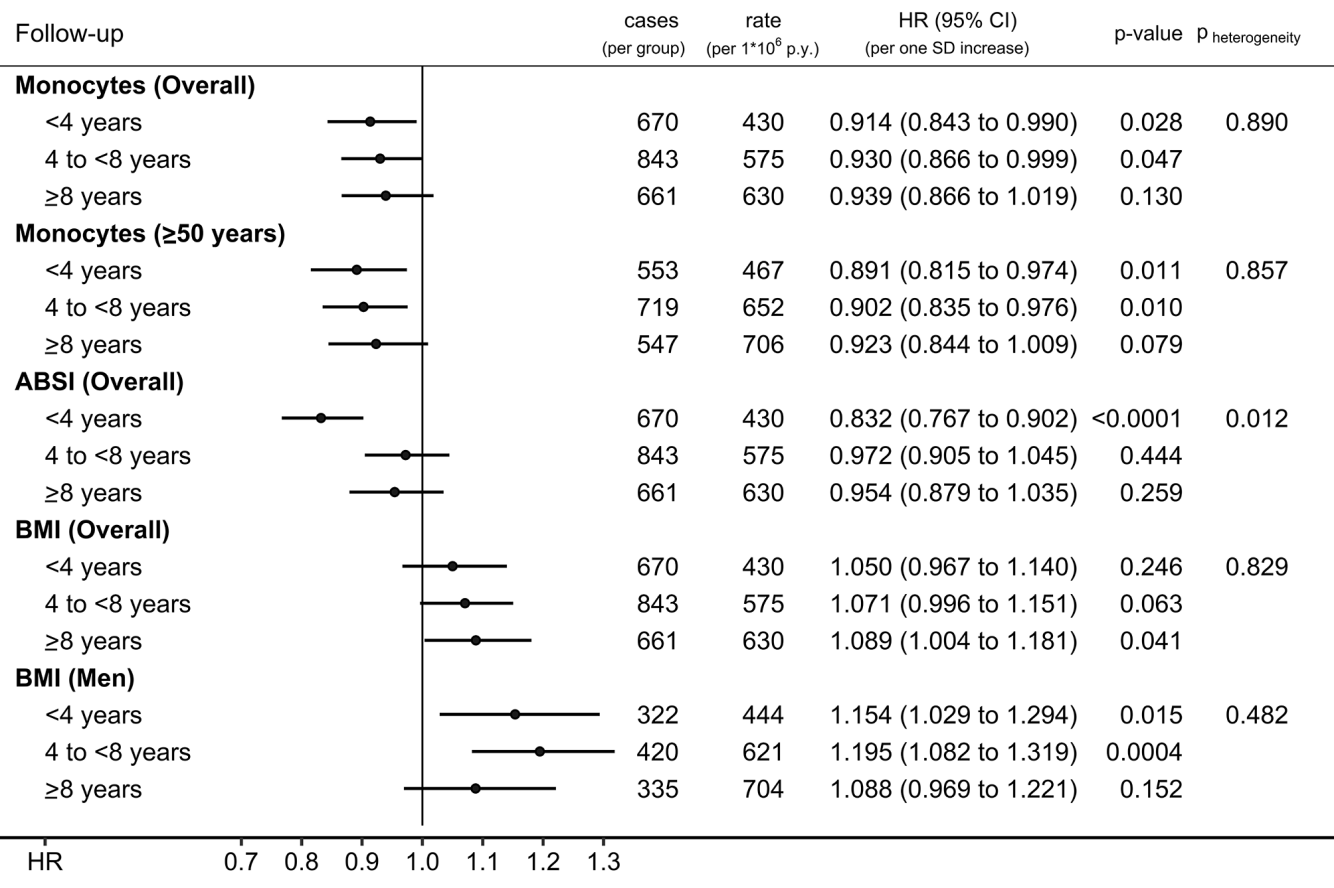
Prospective associations of monocyte count and obesity indices with cutaneous malignant melanoma according to time to diagnosis. **ABSI** – a body shape index; **BMI** – body mass index; **cases** – number of cutaneous malignant melanoma cases; **CI** – confidence interval; **rate** – number of cutaneous malignant melanoma cases per 1,000,000 person years of follow-up within the corresponding period; **HR** – hazard ratio; **Monocytes** – monocyte count; **p-value** – Wald test for the individual term; **SD** – standard deviation. Cox proportional hazards models including jointly monocyte count, ABSI, and BMI as exposures (sex-specific z-scores, value minus mean divided by SD), with mean (SD) for monocyte count: 0.436 (0.144) for women, 0.513 (0.169) for men (unit *10^9^/L); mean (SD) for ABSI: 73.779 (4.953) for women, 79.756 (4.107) for men; and mean (SD) for BMI: 26.917 (4.779) for women, 27.804 (4.037) for men (unit kg/m^2^). Cohort entry was considered the date at the beginning of the corresponding examined follow-up period and exit the earliest of the date at diagnosis of the first primary incident cancer, or death, or the end of the corresponding examined follow-up period, or the end of cancer follow-up (396,450 participants with follow-up < 4 years; 379,446 with follow-up 4 to < 8 years; 352,986 with follow-up ≥ 8 years). All models were stratified by age at recruitment, region, sex, and for analyses including women, menopausal status, and hormone replacement therapy use, and were adjusted for height (sex-specific z-scores), smoking status and intensity, alcohol consumption, Townsend deprivation index, family history of cancer, use of antihypertensive drugs, and sun-exposure-related factors (skin colour, ease of skin tanning, hair colour, childhood sunburns, solarium use, sun/UV protection, and time spent outdoors in summer). **p**_**heterogeneity**_ – p-value obtained with the data augmentation method of Lunn and McNeil [16] for the comparison of HR estimates between the specified periods according to follow-up time

An inverse association of monocyte count with CMM was maintained with no material attenuation to at least 8 years prior to diagnosis in participants overall and in participants aged ≥ 50 years at recruitment (Fig. 4). A positive association of BMI with CMM was also largely retained to at least 8 years prior to diagnosis in participants overall and in men. The inverse association with ABSI, however, was observed only up to 4 years prior to diagnosis and not for longer follow-up time (p_heterogeneity_=0.012 for participants overall).

## Discussion

In this study, monocyte count was associated prospectively inversely with CMM for at least 8 years prior to diagnosis but there was little evidence for associations with neutrophil or lymphocyte count. Our findings are thus compatible with a specific involvement of monocyte-related mechanisms and not of chronic inflammation in melanoma development, in agreement with a previously reported null association with C-reactive protein [17]. In support, animal studies have shown that inflammatory monocytes inhibit melanoma proliferation and dissemination via formation of reactive oxygen species [18], while in patients with CMM, monocytes have limited ability to differentiate and mature to macrophages [19, 20], and show poor response to phagocytic stimuli [21]. In addition, aging is associated with mitochondrial dysfunction in monocytes [22] and impaired monocyte phagocytosis [23], which may be compensated for by higher monocyte count, potentially explaining the more prominent inverse association with monocyte count in older individuals observed in our study. Previous studies examining monocyte count, however, are limited to the time of CMM diagnosis and have shown, on the contrary, worse survival for high monocyte count [24–26]. This is compatible with melanoma cells releasing chemokines to attract infiltrating monocytes, which promote their survival [27, 28], and collaborating with monocytes to produce potent angiogenic factors, which facilitate their metastasis [29]. Thus, considering the dual pro-oncogenic and anti-oncogenic properties of monocytes, future studies would need to clarify the differential contributions of monocyte count and function to melanoma development and progression, as these are likely to influence the success of immunotherapy.

We have corroborated, with a larger number of cases, an inverse association of ABSI with CMM [2, 30]. Although this was limited to 4 years prior to diagnosis and may thus reflect reverse causality, it is in agreement with previously reported longer survival in CMM patients with larger visceral adipose tissue (VAT) [31]. Nevertheless, inverse associations of abdominal size with CMM are puzzling because large VAT is a hallmark of hyperactivity of the hypothalamus-pituitary-adrenal (HPA) axis and glucocorticoid excess [32, 33]. At the same time, melanoma cells produce HPA axis factors [34–39], mice bearing metastatic melanoma and patients with CMM have higher adrenocorticotropic hormone and glucocorticoid levels [40, 41], and malignant melanoma promotes its own progression via activation of a high affinity glucocorticoid receptor (GR) [42, 43]. In accordance, disruption of the HPA axis and GR activity in animal models and cell lines hinders, while glucocorticoid administration promotes melanoma growth [40, 44, 45]. Considering the above, a positive association of abdominal size with melanoma development and progression would be expected. A potential explanation of this discrepancy could be lower glucocorticoid sensitivity of melanoma cells and a correspondingly slower melanoma progression in individuals with GR polymorphisms conferring glucocorticoid resistance and lower VAT accumulation [46]. Glucocorticoid resistance may also explain why the inverse association with ABSI was not observed in post-menopausal women, which have low oestrogen levels. Correspondingly, animal models have shown that oestrogen loss leads to high levels of follicle stimulating hormone, which contributes to increased GR sensitivity and larger VAT [47], potentially hindering an inverse association with ABSI. Notably, only participants with low ABSI showed an inverse association of monocyte count with CMM. This may be due to altered monocyte function in individuals with abdominal obesity, for whom glucocorticoid suppression of interleukin-6 (IL-6) production by monocytes is reduced [48], and they may thus have more pro-tumorigenic monocytes, given that glucocorticoid stimulated IL-6 production in melanoma cells facilitates metastatic growth [40].

We have further corroborated, with a larger number of cases, a positive association of BMI with CMM in men [2, 49], which was retained to at least 8 years prior to diagnosis and is thus compatible with a mechanistic involvement of obesity-related factors in melanoma development. These are less likely to be inflammatory, given the null associations with neutrophil and lymphocyte counts and the inverse association with ABSI, and more likely to be related to subcutaneous adipose tissue, with leptin and oestrogens as the most prominent candidates [50, 51]. In agreement, CMM patients have higher circulating leptin levels at diagnosis [52] and melanoma tumour growth is higher in obese mice with high leptin levels and lower in lean leptin-deficient mice with restricted diet [53]. On the other hand, oestradiol and oestrogen receptor β (ERβ) agonists inhibit melanoma cell proliferation [54, 55] and ERβ, which is the main subtype in melanocytes, is reduced in melanoma cells and is lowest in invasive and metastatic melanoma [56, 57]. Oestrogen action via the G protein-coupled oestrogen receptor (GPER) also favours a more differentiated cell state and suppresses melanoma progression [58]. Thus, higher sensitivity to oestrogens in women, potentially counterbalancing the pro-oncogenic properties of leptin, may explain the more favourable melanoma outcomes in women compared to men [59] and the null association with BMI in women. A prevailing oestrogen over leptin action on monocyte function may also explain the stronger inverse association of monocyte count with CMM in overweight and obese individuals observed in our study. While leptin receptor expression in monocytes is highest among leucocyte subtypes, and leptin induces monocyte proliferation and IL-6 inflammatory responses [60], thus favouring a pro-tumorigenic phenotype, oestrogens acting via ERα and GPER inhibit IL-6 inflammatory responses and induce an anti-inflammatory profile [61], thus facilitating the tumour suppressing properties of monocytes.

Although smoking is associated inversely with CMM [62] and positively with monocyte count and abdominal obesity [63, 64], there was little evidence in our study for residual confounding by smoking, or for heterogeneity according to smoking status. There was, however, only a limited number of CMM cases in smokers and the positive association with BMI was noted only in former and current smokers.

A major strength of our study is the prospective cohort design with available leucocyte subtype counts and a sizable number of incident CMM cases, which allowed us to examine heterogeneity by sex, age, menopausal status, and time to diagnosis. Standardised anthropometric measurements avoided bias from self-reporting of weight and abdominal size. A clear limitation of our study, however, is the lack of information about monocyte function, HPA axis status, and steroid receptor expression and sensitivity. Individuals younger than 40 and older than 70 years were not available either. We were limited to examining individuals with white ancestry because participants from other ethnic backgrounds were few. We could not examine associations with VAT because imaging body composition measurements were assessed only for 10% of participants some 7 years after the haematological parameters. We could not account for changes during follow-up, as exposures and confounders were assessed only at recruitment. Last, UK Biobank participants have a healthier lifestyle compared to the general population [65] and this may limit the range of the examined exposures.

## Conclusions

Monocyte count and ABSI were both associated inversely with the risk of developing CMM, independent of each other, but only the inverse association with monocyte count was maintained prospectively and likely reflects a contribution of monocyte/macrophage dysfunction to melanoma development, while the inverse association with ABSI was confined close to diagnosis and may reflect reverse causality or may be related to glucocorticoid resistance. A positive association of BMI with CMM in men likely reflects the influence of factors related to subcutaneous adipose tissue such as leptin and not chronic inflammation.

### Electronic supplementary material

Below is the link to the electronic supplementary material.

Supplementary Material 1

Supplementary Material 2

## Data Availability

The dataset analysed in the current study was used under license and cannot be made freely available in a public repository or obtained from the authors due to restrictions related to privacy regulations and informed consent of the participants. Access to the data, however, can be obtained by *bona fide* researchers from UK Biobank, subject to approval of the research project and a material transfer agreement. For information on how to apply for gaining access to UK Biobank data, please follow the instructions at https://www.ukbiobank.ac.uk/enable-your-research. Further queries related to the data could be addressed to the corresponding author Dr Sofia Christakoudi s.christakoudi@imperial.ac.uk.

## Abbreviations

ABSI: A body shape index
ACTH: Adrenocorticotropic hormone
BMI: Body mass index
CI: Confidence interval
CRH: Corticotropin releasing hormone
ER: Oestrogen receptor
GR: Glucocorticoid receptor
HPA axis: Hypothalamus pituitary adrenal axis
HR: Hazard ratio
ICD10: 10^th^ revision of the International Statistical Classification of Diseases
IL-6: Interleukin-6
MP: Menopause
SD: Standard deviation
UK: United Kingdom
VAT: Visceral adipose tissue
