# Supplementary material for: Prospective associations of leucocyte subtypes and obesity with the risk of developing cutaneous malignant melanoma in the UK Biobank cohort
Source: BMC Cancer. 2024 May 23;24:625. doi: 10.1186/s12885-024-12344-0 (PMC11112846; doi:10.1186/s12885-024-12344-0)
Supplement: Supplementary file 1 — Supplementary Material 1 [file 12885_2024_12344_MOESM1_ESM.pdf]

Prospective associations of leucocyte subtypes and obesity with the risk of developing cutaneous malignant melanoma in the UK Biobank cohort

Sofia Christakoudi, Konstantinos K. Tsilidis, Elio Riboli

Supplementary Figure

Supplementary Figure S1 Directed acyclic graph of the relationships of candidate covariates with the exposures and the outcome 2

Supplementary Tables

Supplementary Table S1 Rationale for selection of candidate covariates 3

Supplementary Table S2 Associations of candidate covariates with the exposures and the outcome… .... (separate file)

Supplementary Table S3 Characteristics of study participants 5

Supplementary Table S4 Comparison of the main analyses with models using multiple imputations for missing values... 8

References………………..……………………………………………………………………………9

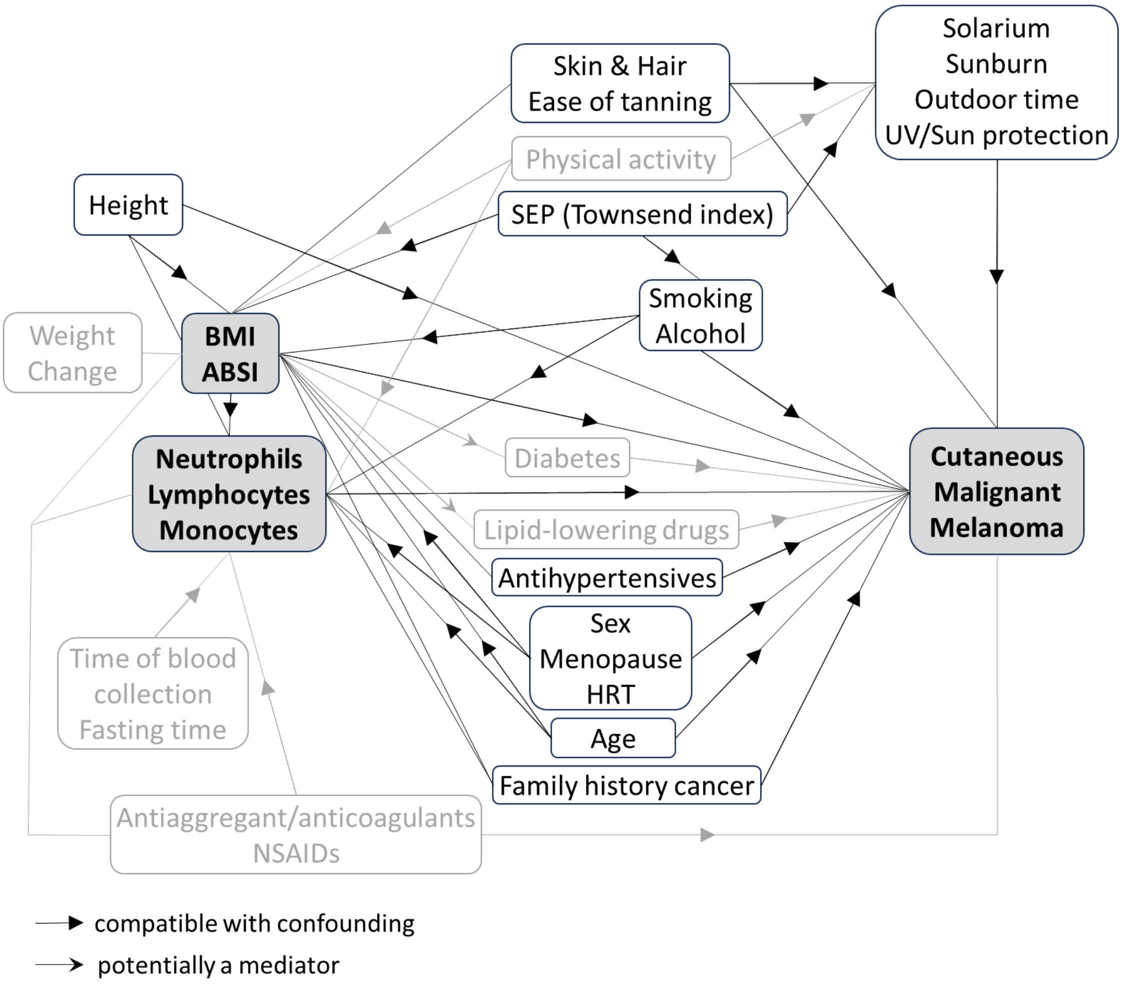

Supplementary Figure S1 Directed acyclic graph of the relationships of candidate covariates with the exposures and the outcome

ABSI – a body shape index; **BMI** – body mass index; **HRT** – hormone replacement therapy; **NSAIDs** – non-steroidal anti-inflammatory drugs; **SEP** – socio-economic position.

Candidate covariates were selected *a priori* based on literature reports of their associations with the exposures and the outcome (**Supplementary Table S1**).

Supplementary Table S1 Rationale for selection of candidate covariates

Factor	Exposures (BMI, ABSI, Leucocytes)	Outcome (CMM)
Height	To remove residual correlations for allometric anthropometric indices, as discussed in Supplementary Methods of [13].	Higher risk for tall [66].
Sex	Sex differences in body composition [67], and leucocyte count [8].	Higher risk in men [68].
HRT	Higher use in post and peri-menopausal women, which have higher BMI and ABSI [12].	Higher risk for HRT users [69].
Weight change	Would reflect different BMI trajectories.	Lower risk for weight loss in men [70].
Smoking	Higher leucocyte counts [64] and abdominal obesity [63] in smokers.	Lower risk in smokers [62].
Alcohol	Alterations in relative blood cell counts, with a shift towards a memory T cell phenotype, as well as an inhibition of the cytoskeleton reorganisation of monocytes [71].	Positive association in men not in women [72].
Physical activity	Lower leucocyte counts and inflammatory markers with physical activity [73].	Positive associations in cohort studies, potentially related to UV exposure [74].
Socio-economic position	Higher risk of abdominal obesity for low SEP, as a potential source of chronic stress [63], but also modifications of monocyte function [75].	Higher risk for higher income [76].
Diabetes	Associated with abdominal obesity as part of the metabolic syndrome.	Higher risk due to phototoxicity of anti-diabetic drugs [77].
Lipid-lowering drugs	May affect leucocyte function due to anti-inflammatory properties, as have been associated with lower C-reactive protein [78].	Higher risk with use of lipophilic statins [79].
Anti-hypertensive drugs	Use would be associated with abdominal obesity, as obesity and hypertension are both are part of the metabolic syndrome.	Higher risk due to phototoxicity [77].
NSAIDs	May affect leucocyte function due to anti-inflammatory properties.	If inflammation is relevant, may contribute to lower risk, but may also contribute to higher risk due to phototoxicity [77].
Antiaggregant/ anticoagulants	Could affect leucocyte function because platelets are involved in immune inflammation [80].	Lower risk with aspirin use in individuals with uncontrolled hypertension [81].
Skin & Hair	The melanocortin pathway, which is relevant to melanoma, is also involved in energy homeostasis [82] and defects in proopiomelanocortin (POMC) synthesis have been associated with red hair, as well as severe obesity [83].	Higher risk for fair hair and white skin [84].

**Factor**	**Exposures (BMI, ABSI, Leucocytes)**	**Outcome (CMM)**
UV exposure	Could be related indirectly to obesity and inflammation due to different behavioural patterns in individuals with different SEP.	Major risk factor [84], although no strong evidence for association with moderate/responsible solarium use [85].
Sun protection	Could be related indirectly to obesity and inflammation due to different behavioural patterns in individuals with different SEP.	Higher risk before 1980s and no evidence for protection [86].
Fasting time & Time of blood collection	Lymphocyte count decreases while neutrophil count increases after food intake [87]. Leucocyte counts are subject to diurnal variation, with higher levels towards the evening [88].	May improve precision of risk estimates, as a way of standardising the conditions of blood collection for leucocyte counts (time of day and fasting time).

ABSI – a body shape index; **BMI** – body mass index; **CMM** – cutaneous malignant melanoma; **HRT** – hormone replacement therapy; **NSAIDs** – non-steroidal anti-inflammatory drugs; **SEP** – socio-economic position.

A directed acyclic graph of the relationships of candidate covariates with the exposures and the outcome is shown in **Supplementary Figure S1**. Pairwise associations of candidate covariates with the exposure and the outcome are shown in **Supplementary Table S2** (separate file).

Supplementary Table S3 Characteristics of study participants

	Overall	Women	Men	Pre-MP	Post-MP	Men <50 years	Men ≥50 years
Cohort	398,450	212,295 (53.3)	186,155 (46.7)	51,520 (27.3)	137,046 (72.7)	43,408 (23.3)	142,747 (76.7)
**Anthropometry**: mean (SD)							
Height: cm	168.8 (9.3)	162.7 (6.2)	175.9 (6.8)	164.3 (6.2)	162.0 (6.1)	177.6 (6.8)	175.4 (6.7)
**BMI groups**: n (%)							
Normal weight (18.5 to <25)	131,737 (33.1)	85,404 (40.2)	46,333 (24.9)	24,909 (48.3)	52,438 (38.3)	12,049 (27.8)	34,284 (24.0)
Overweight (25 to <30)	171,824 (43.1)	79,011 (37.2)	92,813 (49.9)	16,851 (32.7)	53,142 (38.8)	21,295 (49.1)	71,518 (50.1)
Obese (30 to <45)	94,889 (23.8)	47,880 (22.6)	47,009 (25.3)	9760 (18.9)	31,466 (23.0)	10,064 (23.2)	36,945 (25.9)
**Smoking status**: n (%)							
Never smoked	156,704 (39.3)	92,786 (43.7)	63,918 (34.3)	23,888 (46.4)	58,600 (42.8)	17,480 (40.3)	46,438 (32.5)
Just tried	59,265 (14.9)	32,312 (15.2)	26,953 (14.5)	8873 (17.2)	19,889 (14.5)	7945 (18.3)	19,008 (13.3)
Former occasional	46,282 (11.6)	26,002 (12.2)	20,280 (10.9)	5955 (11.6)	17,275 (12.6)	4095 (9.4)	16,185 (11.3)
Former regular: quit ≥20 years	46,416 (11.6)	20,127 (9.5)	26,289 (14.1)	1807 (3.5)	16,241 (11.9)	1069 (2.5)	25,220 (17.7)
Former regular: quit ≥10 years	21,726 (5.5)	9960 (4.7)	11,766 (6.3)	2621 (5.1)	6217 (4.5)	2296 (5.3)	9470 (6.6)
Former regular: quit <10 years	25,455 (6.4)	11,837 (5.6)	13,618 (7.3)	3186 (6.2)	7278 (5.3)	3616 (8.3)	10,002 (7.0)
Former regular: quit missing	391 (0.1)	196 (0.1)	195 (0.1)	25 (<0.1)	153 (0.1)	21 (<0.1)	174 (0.1)
Current occasional	10,707 (2.7)	4362 (2.1)	6345 (3.4)	1507 (2.9)	2335 (1.7)	2072 (4.8)	4273 (3.0)
Current regular: ≤10 cigarettes/day	10,164 (2.6)	5806 (2.7)	4358 (2.3)	1653 (3.2)	3466 (2.5)	1442 (3.3)	2916 (2.0)
Current regular: >10 cigarettes/day	19,033 (4.8)	8119 (3.8)	10,914 (5.9)	1888 (3.7)	5030 (3.7)	3190 (7.3)	7724 (5.4)
Current regular: cigarettes/day missing	1019 (0.3)	109 (0.1)	910 (0.5)	18 (<0.1)	82 (0.1)	104 (0.2)	806 (0.6)
Missing	1288 (0.3)	679 (0.3)	609 (0.3)	99 (0.2)	480 (0.4)	78 (0.2)	531 (0.4)
**Alcohol consumption**: n (%)							
≤3 times / month	111,809 (28.1)	74,035 (34.9)	37,774 (20.3)	16,327 (31.7)	48614 (35.5)	9828 (22.6)	27,946 (19.6)
≤4 times / week	202,072 (50.7)	102,522 (48.3)	99,550 (53.5)	27,703 (53.8)	63,802 (46.6)	25,242 (58.2)	74,308 (52.1)
Daily	84,298 (21.2)	35,612 (16.8)	48,686 (26.2)	7465 (14.5)	24,552 (17.9)	8292 (19.1)	40,394 (28.3)
Missing	271 (0.1)	126 (0.1)	145 (0.1)	25 (<0.1)	78 (0.1)	46 (0.1)	99 (0.1)
**Townsend index**							
Median (IQR)	-2.27 (3.93)	-2.28 (3.85)	-2.26 (4.02)	-2.09 (4.09)	-2.36 (3.74)	-1.86 (4.47)	-2.37 (3.87)
Missing: n (%)	466 (0.1)	236 (0.1)	230 (0.1)	73 (0.1)	137 (0.1)	75 (0.2)	155 (0.1)
**Family history**: n (%)							
No cancer	257,663 (64.7)	136,556 (64.3)	121,107 (65.1)	36,553 (70.9)	84,884 (61.9)	31,246 (72.0)	89,861 (63.0)
Breast/ bowel/ prostate/ lung cancer	140,787 (35.3)	75,739 (35.7)	65,048 (34.9)	14,967 (29.1)	52,162 (38.1)	12,162 (28.0)	52,886 (37.0)
**Antihypertensive drugs use**: n (%)							
Yes	79,439 (19.9)	35,272 (16.6)	44,167 (23.7)	2559 (5.0)	28,134 (20.5)	3042 (7.0)	41,125 (28.8)
Missing	2434 (0.6)	839 (0.4)	1595 (0.9)	240 (0.5)	453 (0.3)	478 (1.1)	1117 (0.8)

	Overall	Women	Men	Pre-MP	Post-MP	Men <50 years	Men ≥50 years
**Skin colour**: n (%)							
Very fair	30,841 (7.7)	19,133 (9.0)	11,708 (6.3)	5419 (10.5)	11,531 (8.4)	3104 (7.2)	8604 (6.0)
Fair	279,930 (70.3)	146,809 (69.2)	133,121 (71.5)	34,670 (67.3)	95,886 (70.0)	29,467 (67.9)	103,654 (72.6)
Dark	82,769 (20.8)	44,304 (20.9)	38,465 (20.7)	11,144 (21.6)	28,158 (20.5)	10,213 (23.5)	28,252 (19.8)
Missing	4910 (1.2)	2049 (1.0)	2861 (1.5)	287 (0.6)	1471 (1.1)	624 (1.4)	2237 (1.6)
**Ease of skin tanning**: n (%)							
Get very tanned	81,870 (20.5)	33,368 (15.7)	48,502 (26.1)	8100 (15.7)	21,420 (15.6)	10,262 (23.6)	38,240 (26.8)
Get moderately tanned	158,731 (39.8)	81,356 (38.3)	77,375 (41.6)	19,449 (37.8)	53,001 (38.7)	17,300 (39.9)	60,075 (42.1)
Get mildly/occasionally tanned	83,200 (20.9)	51,954 (24.5)	31,246 (16.8)	13,536 (26.3)	32,774 (23.9)	8795 (20.3)	22,451 (15.7)
Never tan/ only burn	67,091 (16.8)	41,034 (19.3)	26,057 (14.0)	9663 (18.8)	26,563 (19.4)	6409 (14.8)	19,648 (13.8)
Missing	7558 (1.9)	4583 (2.2)	2975 (1.6)	772 (1.5)	3288 (2.4)	642 (1.5)	2333 (1.6)
**Hair colour**: n (%)							
Blond or red	61,654 (15.5)	36,909 (17.4)	24,745 (13.3)	9224 (17.9)	23,495 (17.1)	5898 (13.6)	18,847 (13.2)
Light brown	160,653 (40.3)	88,495 (41.7)	72,158 (38.8)	21,088 (40.9)	57,546 (42.0)	15,587 (35.9)	56,571 (39.6)
Dark	175,394 (44.0)	86,730 (40.9)	88,664 (47.6)	21,174 (41.1)	55,913 (40.8)	21,785 (50.2)	66,879 (46.9)
Missing	749 (0.2)	161 (0.1)	588 (0.3)	34 (0.1)	92 (0.1)	138 (0.3)	450 (0.3)
**Solarium use**: n (%)							
Never use	356,954 (89.6)	186,827 (88.0)	170,127 (91.4)	42,937 (83.3)	123,508 (90.1)	38402 (88.5)	131,725 (92.3)
Ever use	38,961 (9.8)	23,986 (11.3)	14,975 (8.0)	8224 (16.0)	12,648 (9.2)	4732 (10.9)	10,243 (7.2)
Missing	2535 (0.6)	1482 (0.7)	1053 (0.6)	359 (0.7)	890 (0.6)	274 (0.6)	779 (0.5)
**Sunburn in childhood**: n (%)							
Never burned	156,283 (39.2)	91,066 (42.9)	65,217 (35.0)	18,291 (35.5)	62,838 (45.9)	12,562 (28.9)	52,655 (36.9)
Ever burned	143,647 (36.1)	71,054 (33.5)	72,593 (39.0)	22,935 (44.5)	40,268 (29.4)	21,345 (49.2)	51,248 (35.9)
Missing [#]	98,520 (24.7)	50,175 (23.6)	48,345 (26.0)	10,294 (20.0)	33,940 (24.8)	9501 (21.9)	38,844 (27.2)
**Time spent outdoors in summer**: n (%)							
≤ 3 hours a day	197,278 (49.5)	112,355 (52.9)	84,923 (45.6)	32,109 (62.3)	67,824 (49.5)	24,348 (56.1)	60,575 (42.4)
> 3 hours a day	179,757 (45.1)	86,345 (40.7)	93,412 (50.2)	16,781 (32.6)	60,031 (43.8)	17,178 (39.6)	76,234 (53.4)
Missing [#]	21,415 (5.4)	13,595 (6.4)	7820 (4.2)	2630 (5.1)	9191 (6.7)	1882 (4.3)	5938 (4.2)
**Sun / UV protection**: n (%)							
Never / rarely	33,829 (8.5)	8553 (4.0)	25,276 (13.6)	1690 (3.3)	5887 (4.3)	4828 (11.1)	20,448 (14.3)
Sometimes	134,507 (33.8)	58,326 (27.5)	76,181 (40.9)	13,235 (25.7)	38,755 (28.3)	17,149 (39.5)	59,032 (41.4)
Most of the time	145,919 (36.6)	86,797 (40.9)	59,122 (31.8)	22,948 (44.5)	54,356 (39.7)	15,503 (35.7)	43,619 (30.6)
Always / do not go out in sunshine	83,965 (21.1)	58,544 (27.6)	25,421 (13.7)	13,637 (26.5)	38,006 (27.7)	5876 (13.5)	19,545 (13.7)
Missing	230 (0.1)	75 (<0.1)	155 (0.1)	10 (<0.1)	42 (<0.1)	52 (0.1)	103 (0.1)

	Overall	Women	Men	Pre-MP	Post-MP	Men <50 years	Men ≥50 years
**Age at recruitment:** n (%)							
40 to <45 years	41,070 (10.3)	21,723 (10.2)	19,347 (10.4)	19,706 (38.2)	647 (0.5)	19,347 (44.6)	
45 to <50 years	53,064 (13.3)	29,003 (13.7)	24,061 (12.9)	21,152 (41.1)	3874 (2.8)	24,061 (55.4)	
50 to <55 years	61,493 (15.4)	33,916 (16.0)	27,577 (14.8)	9305 (18.1)	18,347 (13.4)		27,577 (19.3)
55 to <60 years	73,219 (18.4)	39,943 (18.8)	33,276 (17.9)	890 (1.7)	34,967 (25.5)		33,276 (23.3)
60 to <65 years	96,749 (24.3)	51,390 (24.2)	45,359 (24.4)	195 (0.4)	46,639 (34.0)		45,359 (31.8)
65 to 70 years	72,855 (18.3)	36,320 (17.1)	36,535 (19.6)	272 (0.5)	32,572 (23.8)		36,535 (25.6)
**Region**: n (%)							
London	46,804 (11.7)	25,379 (12.0)	21,425 (11.5)	6691 (13.0)	16,433 (12.0)	5095 (11.7)	16,330 (11.4)
North-West	61,329 (15.4)	31,965 (15.1)	29,364 (15.8)	7191 (14.0)	20,871 (15.2)	6650 (15.3)	22,714 (15.9)
North-East	48,875 (12.3)	26,036 (12.3)	22,839 (12.3)	5916 (11.5)	17,338 (12.7)	5184 (11.9)	17,655 (12.4)
Yorkshire Humber	60,734 (15.2)	32,353 (15.2)	28,381 (15.2)	7559 (14.7)	20,674 (15.1)	6395 (14.7)	21,986 (15.4)
West Midlands	34,684 (8.7)	17,377 (8.2)	17,307 (9.3)	4059 (7.9)	11,479 (8.4)	3929 (9.1)	13,378 (9.4)
East Midlands	27,601 (6.9)	14,708 (6.9)	12,893 (6.9)	3337 (6.5)	9733 (7.1)	2755 (6.3)	10,138 (7.1)
South-East	35,894 (9.0)	19,570 (9.2)	16,324 (8.8)	4804 (9.3)	12,432 (9.1)	3914 (9.0)	12,410 (8.7)
South-West	35,793 (9.0)	19,592 (9.2)	16,201 (8.7)	5394 (10.5)	11,880 (8.7)	4277 (9.9)	11,924 (8.4)
Wales	17,022 (4.3)	9061 (4.3)	7961 (4.3)	2327 (4.5)	5650 (4.1)	1922 (4.4)	6039 (4.2)
Scotland	29,714 (7.5)	16,254 (7.7)	13,460 (7.2)	4242 (8.2)	10,556 (7.7)	3287 (7.6)	10,173 (7.1)
**Menopause-HRT-use**: n (%)							
Pre-menopausal		51,520 (24.3)		51,520 (100)			
Post/Unknown: Never		80,231 (37.8)			67,143 (49.0)		
Post/Unknown: Past		63,050 (29.7)			56,078 (40.9)		
Post/Unknown: Current		17,494 (8.2)			13,825 (10.1)		

HRT – hormone replacement therapy; **IQR** – interquartile range; **MP** – menopause; **n (%)** – number of participants (percentage from total per column); **NSAID** – non-steroidal anti-inflammatory drugs; # – used as a separate category.

Comparisons between women and men, pre-menopausal and post-menopausal women, and men <50 and ≥50 years were performed with unpaired-samples t-test for height, Wilcoxon rank sum (Mann-Whitney) test for Townsend deprivation index, and χ^2-test for categorical variables (after imputation). All differences were significant at p<0.0001, except missing Townsend deprivation index.

For definition of anti-hypertensive medications see Supplementary Methods and Supplementary Table S1 in [8]. For definition of HRT use, see Supplementary Methods and Supplementary Table S1 in [12].

Supplementary Table S4 Comparison of the main analyses with models using multiple imputations for missing values

	Main analysis		MI analysis	
	HR (95% CI)	p-value	HR (95% CI)	p-value
**Neutrophil count**				
Overall	1.011 (0.966 to 1.058)	0.627	1.011 (0.966 to 1.058)	0.642
Women	1.028 (0.965 to 1.095)	0.394	1.028 (0.965 to 1.095)	0.398
Men	0.998 (0.935 to 1.066)	0.959	0.997 (0.934 to 1.065)	0.939
**Lymphocyte count**				
Overall	1.011 (0.967 to 1.057)	0.634	1.011 (0.967 to 1.057)	0.629
Women	0.986 (0.926 to 1.051)	0.667	0.986 (0.926 to 1.051)	0.668
Men	1.033 (0.970 to 1.100)	0.310	1.033 (0.971 to 1.100)	0.304
**Monocyte count**				
Overall	0.928 (0.888 to 0.971)	0.001	0.928 (0.888 to 0.971)	0.001
Women	0.956 (0.898 to 1.017)	0.153	0.956 (0.898 to 1.017)	0.154
Men	0.898 (0.842 to 0.957)	0.001	0.897 (0.842 to 0.957)	0.001
<50 years	1.051 (0.943 to 1.171)	0.371	1.051 (0.944 to 1.172)	0.364
≥50 years	0.906 (0.862 to 0.951)	<0.0001	0.906 (0.862 to 0.951)	<0.0001
**ABSI**				
Overall	0.922 (0.881 to 0.964)	0.0004	0.921 (0.881 to 0.964)	0.0004
Women	0.923 (0.866 to 0.983)	0.012	0.923 (0.866 to 0.983)	0.012
Men	0.925 (0.867 to 0.986)	0.017	0.923 (0.865 to 0.985)	0.015
**BMI**				
Overall	1.069 (1.022 to 1.118)	0.004	1.068 (1.021 to 1.117)	0.004
Women	0.990 (0.927 to 1.056)	0.753	0.989 (0.927 to 1.056)	0.745
Men	1.148 (1.078 to 1.222)	<0.0001	1.147 (1.077 to 1.221)	<0.0001

ABSI – a body shape index; **BMI** – body mass index; **MI** – multiple imputations.

All models included ABSI, BMI, and one of the leucocyte counts on a standardised continuous scale (sex-specific z-scores, value minus mean divided by standard deviation), and were stratified by age at recruitment, region, sex, and for analyses including women, menopausal status and hormone replacement therapy use, and adjusted for height (sex-specific z-scores), smoking status and intensity, alcohol consumption, Townsend deprivation index, family history of cancer, use of anti-hypertensive drugs, and sun-exposure-related factors (skin colour, ease of skin tanning, hair colour, sunburns in childhood, solarium use, sun/UV protection, and time spent outdoors in summer) (Model 4 in Figures 1, 2, and 3). Estimates for monocyte count, ABSI, and BMI were derived from the same model.

We performed multiple sequential imputations with chained equations (function *mi impute* in Stata 13, m=5 imputed datasets) for skin colour, ease of skin tanning, sunburns in childhood, and time spent outdoors in summer (which had missingness >1%) using multinomial logistic regression models stratified by region and including sex, age at recruitment, smoking status and intensity, Townsend deprivation index, and for all variables except time spent outdoors in summer also hair colour. We derived the estimates of coefficients and standard errors with Rubin's combination rules (function *mi estimate* in Stata 13) [15].

References

References cited in the main document:

8. Christakoudi S, Riboli E, Evangelou E, Tsilidis KK. Associations of body shape index (ABSI) and hip index with liver, metabolic, and inflammatory biomarkers in the UK Biobank cohort. *Sci Rep*. 2022;12(1):8812. doi:10.1038/s41598-022-12284-4.

12. Christakoudi S, Riboli E, Evangelou E, Tsilidis KK. Associations of body shape phenotypes with sex steroids and their binding proteins in the UK Biobank cohort. *Sci Rep*. 2022;12(1):10774. doi:10.1038/s41598-022-14439-9.

13. Christakoudi S, Tsilidis KK, Evangelou E, Riboli E. Interactions of platelets with obesity in relation to lung cancer risk in the UK Biobank cohort. *Respir Res*. 2023;24(1):249. doi:10.1186/s12931-023-02561-9.

15. STATA Multiple-Imputation Reference Manual Release 13. *URL: https://www.stata.com/manuals13/mi.pdf (Accessed 31/10/2023)*.

62. Thompson JF, Friedman EB. The intriguing association between smoking and reduced melanoma risk. *Br J Dermatol*. 2020;182(5):1080. doi:10.1111/bjd.18728.

63. Bjorntorp P. Visceral obesity: a "civilization syndrome". *Obes Res*. 1993;1(3):206-22. doi:10.1002/j.1550-8528.1993.tb00614.x.

64. Smith MR, Kinmonth AL, Luben RN, Bingham S, Day NE, Wareham NJ et al. Smoking status and differential white cell count in men and women in the EPIC-Norfolk population. *Atherosclerosis*. 2003;169(2):331-7. doi:10.1016/s0021-9150(03)00200-4.

Supplementary references:

66. Dusingize JC, Olsen CM, An J, Pandeya N, Law MH, Thompson BS et al. Body mass index and height and risk of cutaneous melanoma: Mendelian randomization analyses. *Int J Epidemiol*. 2020;49(4):1236-45. doi:10.1093/ije/dyaa009.

67. Christakoudi S, Tsilidis KK, Evangelou E, Riboli E. Association of body-shape phenotypes with imaging measures of body composition in the UK Biobank cohort: relevance to colon cancer risk. *BMC cancer*. 2021;21(1):1106. doi:10.1186/s12885-021-08820-6.

68. Buja A, Rugge M, Damiani G, Zorzi M, De Toni C, Vecchiato A et al. Sex Differences in Cutaneous Melanoma: Incidence, Clinicopathological Profile, Survival, and Costs. *J Womens Health (Larchmt)*. 2022;31(7):1012-9. doi:10.1089/jwh.2021.0223.

69. Sun Q, Sun H, Cong L, Zheng Y, Wu N, Cong X. Effects of Exogenous Hormones and Reproductive Factors on Female Melanoma: A Meta-Analysis. *Clin Epidemiol*. 2020;12:1183-203. doi:10.2147/clep.S273566.

70. Stenehjem JS, Veierød MB, Nilsen LT, Ghiasvand R, Johnsen B, Grimsrud TK et al. Anthropometric factors and cutaneous melanoma: Prospective data from the population-based Janus Cohort. *Int J Cancer*. 2018;142(4):681-90. doi:10.1002/ijc.31086.

71. Shalchi-Amirkhiz P, Bensch T, Proschmann U, Stock AK, Ziemssen T, Akgün K. Pilot study on the influence of acute alcohol exposure on biophysical parameters of leukocytes. *Front Mol Biosci*. 2023;10:1243155. doi:10.3389/fmolb.2023.1243155.

72. Mahamat-Saleh Y, Al-Rahmoun M, Severi G, Ghiasvand R, Veierod MB, Caini S et al. Baseline and lifetime alcohol consumption and risk of skin cancer in the European Prospective Investigation into Cancer and Nutrition cohort (EPIC). *Int J Cancer*. 2023;152(3):348-62. doi:10.1002/ijc.34253.

73. Klasson CL, Sadhir S, Pontzer H. Daily physical activity is negatively associated with thyroid hormone levels, inflammation, and immune system markers among men and women in the NHANES dataset. *PLoS One*. 2022;17(7):e0270221. doi:10.1371/journal.pone.0270221.

74. Behrens G, Niedermaier T, Berneburg M, Schmid D, Leitzmann MF. Physical activity, cardiorespiratory fitness and risk of cutaneous malignant melanoma: Systematic review and meta-analysis. *PLoS One*. 2018;13(10):e0206087. doi:10.1371/journal.pone.0206087.

75. Baumer Y, Pita MA, Turner BS, Baez AS, Ortiz-Whittingham LR, Gutierrez-Huerta CA et al. Neighborhood socioeconomic deprivation and individual-level socioeconomic status are associated with dopamine-mediated changes to monocyte subset CCR2 expression via a cAMP-dependent pathway. *Brain Behav Immun Health*. 2023;30:100640. doi:10.1016/j.bbih.2023.100640.

76. Johnson-Obaseki SE, Labajian V, Corsten MJ, McDonald JT. Incidence of cutaneous malignant melanoma by socioeconomic status in Canada: 1992-2006. *J Otolaryngol Head Neck Surg*. 2015;44:53. doi:10.1186/s40463-015-0107-1.

77. Hofmann GA, Weber B. Drug-induced photosensitivity: culprit drugs, potential mechanisms and clinical consequences. *J Dtsch Dermatol Ges*. 2021;19(1):19-29. doi:10.1111/ddg.14314.

78. Kandelouei T, Abbasifard M, Imani D, Aslani S, Razi B, Fasihi M et al. Effect of Statins on Serum level of hs-CRP and CRP in Patients with Cardiovascular Diseases: A Systematic

Review and Meta-Analysis of Randomized Controlled Trials. *Mediators Inflamm*. 2022;2022:8732360. doi:10.1155/2022/8732360.

79. Wang D, Dai S, Lou D, Wang T, Wang S, Zheng Z. Association between statins exposure and risk of skin cancer: an updated meta-analysis. *Int J Dermatol*. 2023. doi:10.1111/ijd.16816.

80. Dib PRB, Quirino-Teixeira AC, Merij LB, Pinheiro MBM, Rozini SV, Andrade FB et al. Innate immune receptors in platelets and platelet-leukocyte interactions. *J Leukoc Biol*. 2020;108(4):1157-82. doi:10.1002/jlb.4mr0620-701r.

81. Yan MK, Orchard SG, Adler NR, Wolfe R, McLean C, Rodríguez LM et al. Association between hypertension and cutaneous melanoma, and the effect of aspirin: extended follow-up of a large randomised controlled trial. *Cancer Epidemiol*. 2022;79:102173. doi:10.1016/j.canep.2022.102173.

82. Yeo GSH, Chao DHM, Siegert AM, Koerperich ZM, Ericson MD, Simonds SE et al. The melanocortin pathway and energy homeostasis: From discovery to obesity therapy. *Mol Metab*. 2021;48:101206. doi:10.1016/j.molmet.2021.101206.

83. Ozen S, Aldemir O. Early-onset severe obesity with ACTH deficiency and red hair in a boy: the POMC deficiency. *Genet Couns*. 2012;23(4):493-5.

84. Leiter U, Keim U, Garbe C. Epidemiology of Skin Cancer: Update 2019. *Adv Exp Med Biol*. 2020;1268:123-39. doi:10.1007/978-3-030-46227-7_6.

85. Burgard B, Schöpe J, Holzschuh I, Schiekofer C, Reichrath S, Stefan W et al. Solarium Use and Risk for Malignant Melanoma: Meta-analysis and Evidence-based Medicine Systematic Review. *Anticancer Res*. 2018;38(2):1187-99. doi:10.21873/anticanres.12339.

86. Silva ESD, Tavares R, Paulitsch FDS, Zhang L. Use of sunscreen and risk of melanoma and non-melanoma skin cancer: a systematic review and meta-analysis. *Eur J Dermatol*. 2018;28(2):186-201. doi:10.1684/ejd.2018.3251.

87. Koscielniak BK, Charchut A, Wójcik M, Sztefko K, Tomasik PJ. Impact of Fasting on Complete Blood Count Assayed in Capillary Blood Samples. *Lab Med*. 2017;48(4):357-61. doi:10.1093/labmed/lmx044.

88. Sennels HP, Jørgensen HL, Hansen AL, Goetze JP, Fahrenkrug J. Diurnal variation of hematology parameters in healthy young males: the Bispebjerg study of diurnal variations. *Scand J Clin Lab Invest*. 2011;71(7):532-41. doi:10.3109/00365513.2011.602422.
